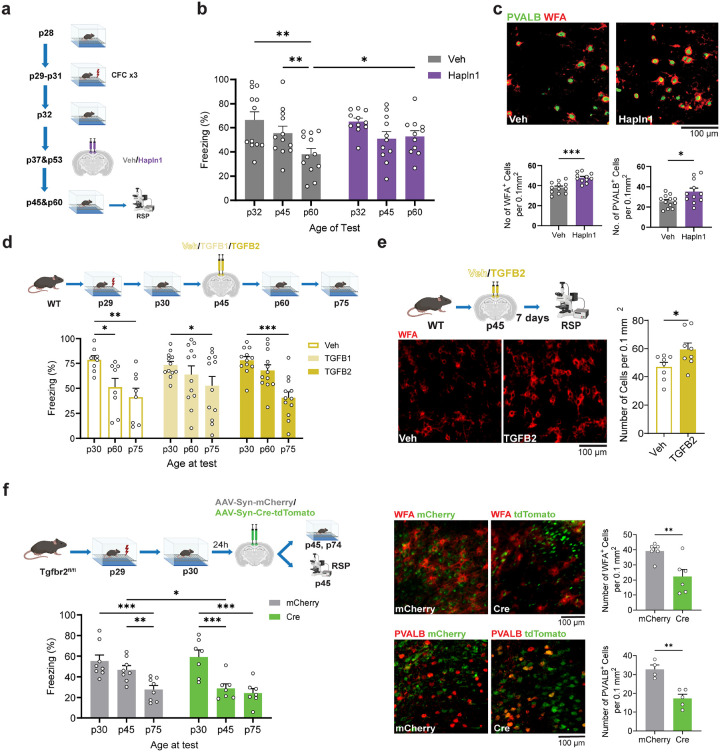# Retrosplenial Cortical Reorganization During Late Adolescence Introduces Instability of Episodic Memory Circuits

**DOI:** 10.21203/rs.3.rs-6864379/v1

**Published:** 2025-09-10

**Authors:** Jelena Radulovic, Hui Zhang, Zorica Petrovic, Elizabeth Wood, Ana Cicvaric, Maayan Krispil-Alon, Vladimir Jovasevic, Kendra Parker, Thomas Bassett, Anna Carboncino, Anita Guedea, Pengfei Yi, Gal Richter-levin, J. Goncalves

**Affiliations:** Albert Einstein College of Medicine; Albert Einstein Medical College; Albert Einstein College of Medicine; Albert Einstein College of Medicine; Albert Einstein College of Medicine; Albert Einstein College of Medicine; Northwestern University; Northwestern University; University of Haifa

## Abstract

Impairments of episodic memory, the capacity to remember and re-live details from one’s past, are common across mental illnesses, but are particularly severe in those with late adolescent onset, such as schizophrenia and major depression. Using mice, we demonstrate that during late adolescence, the retrosplenial cortex (RSP), an established hub of episodic memory and default mode networks, undergoes extensive reorganization, interfering with memory functions and potentially increasing vulnerability to these illnesses. Specifically, we demonstrated that the levels of perineuronal nets (PNN), key PNN constituents, and parvalbumin-positive (PVALB) interneurons established during early adolescence (p30), significantly declined by late adolescence(p60–75). Using context fear conditioning (CFC), we found that these structural, molecular, and cellular changes facilitated the formation of long-lasting and context-specific memories, but at the expense of memories acquired during early adolescence. Interestingly, early adolescent memories spontaneously recovered by middle adulthood but lost context specificity. The observed neurobiological and behavioral changes were attenuated by stabilizing PNN, and exacerbated by disrupting PNN, suggesting that PVALB neuron loss and memory expression deficits were secondary to PNN degradation. These findings showed that despite its superior performance during early adolescence relative to early life, the RSP memory circuit does not show full cortical maturity until late adolescence. In susceptible individuals, the observed dynamics of the extracellular matrix and PVALB neurons could interact with genetic factors, increasing risk for the development of late adolescent psychopathologies.

## Introduction

Episodic memory is a capacity to form long-term memories of unique personal experiences that enable individuals to consciously remember and re-experience past events and situations^1, 2^. In humans, this capacity is emerges in early childhood, showing a linear growth in its spatial, temporal, and item components^3^. The development of the brain in early childhood has been studied extensively, not least because of the well-established “loss” of early childhood memories, a phenomenon known as infantile amnesia. However, as Lüchinger et al. noted^4^, adolescent changes of brain structure and function have rarely been treated as distinct maturational steps despite mounting evidence of extensive brain changes throughout adolescence, especially within cortical areas^5^. Such maturation involves changes of gray matter^6–8^, decrease of low-frequency EEG power^4, 9^, decrease in cortical neuron complexity^10^, and changes of structural connectivity^11^. Understanding the molecular mechanisms underlying these changes, and their impact on episodic memory could provide important insights not only into the dynamics of adolescent memories, but also into vulnerabilities to psychopathologies emerging during this period.

The dynamics of juvenile and adolescent episodic memories related to stressful experiences has been one of the most contested and controversial topics in the memory field because reports of enhanced memory for negative emotional content sharply contrast reports of impaired memory recall and even memory suppression^12, 13^. Findings from rodent memory models, such as CFC and passive avoidance, have been similarly inconclusive with respect to the fate of adolescent memories with reports of memory suppression^14, 15^ contrasting with observations of adult-like memory phenotypes^16, 17^.

At the neurobiological level, episodic memories are viewed as specific patterns of neuronal activity distributed across hippocampal and cortical networks, including the entorhinal, prefrontal, and retrosplenial (RSP) cortices^18–20^. Depending on the stability of memory circuits, the ability to reconstruct these patterns through neuronal reactivation is achieved, among other mechanisms, through rearrangement of the extracellular matrix (ECM) causing build-up of perineuronal nets (PNN) around (predominantly) inhibitory neurons^21, 22^. PNN are believed to stabilize neuronal circuits by several mechanisms, such as limiting access to interfering synaptic inputs and constraining local inhibition^21, 23^. Based on hippocampal studies, it is widely believed that PNN in mice reach adult levels between postnatal days 24–28 (p24-p28), corresponding to early adolescence in humans^16, 24, 25^. The cortical PNN dynamics at later developmental periods, especially with respect to episodic memory functions, is less well understood.

Here, we studied the dynamics of PNN and interneurons from early adolescence to middle adulthood. We focused on RSP because of its direct interaction with the dorsal hippocampus (DH)^26, 27^, its unique, time-independent contribution to memory expression^28–32^, its key role in driving episodic^33, 34^ and default mode^35, 36^ networks, and its established association with episodic memory deficits in adolescent onset psychiatric disorders^37–39^. We demonstrated that PNN and parvalbumin (PVALB)-positive interneurons established during early adolescence undergo RSP-specific downregulation during late adolescence, causing impaired expression of memories acquired prior to these changes. PNN stabilization experiments indicated that both the decrease of PVALB interneuron numbers and memory expression were secondary to PNN degradation. The observed memory deficits were not due to memory loss, as revealed by spontaneous recovery and reinstatement experiments. This suggested that PNN fluctuations did not cause memory degradation or impaired retention, but instead prioritized or restricted access to memories acquired at different developmental periods. Accordingly, access to adolescent memories spontaneously recovered by middle adulthood with the increase of PNN and PVALB interneuron numbers. Interestingly, recovered memories retained specificity for the negative experience, but not for contextual detail. Together, these findings identify late adolescence as a critical period of ECM dynamics introducing instability and, possibly, vulnerability in a cortical area driving key memory networks.

## Materials and methods

### Animals

Wild-type (C57BL/6N) male and female mice were obtained from Envigo (Lafayette, IN, USA). Tgfbr2^fl/fl^ (# 012603)^40^, Vglut1-IRES2-Cre-D(#037512)^41^ and Neurotensin-IRES-tauGFP (NT-GFP; strain *Nts*^*tm1Mom*^/MomJ, #006702)^42^ mice were obtained from Jackson Laboratory (Bar Harbor, ME, USA) and bred in the institutional animal facility. Animals were maintained under 12 h:12 h light:dark cycle with lights on at 07:00, temperature 20–22 °C, humidity 30–60% with ad libitum access to food and water. All procedures were approved by Northwestern University Animal Care and Use Committee (protocols IS00002463 and IS00003359) and Albert Einstein College of Medicine Animal Care and Use Committee (protocols 00001289 and 00001268) in compliance with US National Institutes of Health standards.

### Contextual Fear Conditioning

Contextual fear conditioning (CFC) was performed in automated system (TSE Systems, Berlin, Germany or Med associate, Fairfax, VT, USA). Mice were exposed to Context A (CtxA) for 3 min, followed by a foot shock (0.7 mA, 2 s). On indicated days of test, freezing level of mice to the context were measured in CtxA or Context B (CtxB, different from Context A in shape, floor material, smell, and background noise) for 3 min. In most experiments, mice were tested once a week until p60 or p75, except for a spontaneous recovery experiment, in which mice were tested once a month until p181.

### Contextual discrimination

The contextual discrimination task was performed over 4 days to explicitly determine the ability of mice to discriminate between CtxA and CtxB. On day 1, the mice were allowed to explore CtxA and Context B (CtxB, differ to Context A in shape, floor material, and smell), each for 3 min. On day 2–4, mice were exposed to CtxA for 3 min followed by a tone (30s, 75 dB SPL, 10 kHz, 200 ms pulse) and a foot shock (0.7 mA, 2 s, constant current) sequentially once each day. On the day of test, freezing levels were measured during exposure of mice to CtxA and CtxB for 3 min each. The same tone used on day 2–4 was played at the end of CtxB test for 30 s to measure the freezing level to the auditory cue. Exposures to CtxA and CtxB were always spaced 3 h apart and counterbalanced.

### Memory reinstatement

Memory reinstatement was determined by measuring the effect of early adolescent CtxA CFC on late adolescence CtxB CFC. We subjected the mice to CFC mice CtxA on p29 followed by 2 tests on p30 and p74. On p75 they were exposed to CtxB for 3 min and received a shock reminder (0.7 mA, 2 s) at the end of the exposure. On p76 freezing was determined in CtxA and CtxB for 3 min each, spaced 3 h apart, and counterbalanced. Interference of prior CFC in CtxA with freezing to CtxB and generalization of freezing to CtxA after CFC in CtxB served as indices of CtxA memory retention.

### Environmental enrichment

The memory reinstatement procedure was repeated in both the enriched environment (EE) and control groups, with additional CtxA tests conducted at p44 and p60 After CFC on p29, mice in EE group were placed in EE for on p37 to p60, while control group remain in standard housing. Two identical EE boxes were made of clear polycarbonate, 12 inches high, 45.5 inches long, and 21.5 inches wide, with two filtered air openings in the lid and nontransparent bottom covered with 5 cm of bedding material to prevent fear of heights. The boxes were placed on a stainless-steel rack in a climate-controlled room on a 12 h light/dark cycle. Males and females were placed in separate boxes, and each box contained 6 mice at a time, with three running wheels, three tunnels, three domes, a water and food station. The same layout was used in both boxes throughout the experiment. For behavioral test on p44, mice were briefly moved into standard housing and returned to EE after the test. After p60 mice were returned permanently into standard housing. The control mice were maintained in standard housing during this time.

### Stereotaxic intracranial injection

Mice were anesthetized with isoflurane and placed on a stereotaxic instrument (Model 1900, Kopf Instruments, Tujunga, CA, USA). Viral vectors carrying AAV8-hSyn-mCherry (114472-AAV8, Addgene, gift from Karl Deisseroth), AAV8-hSyn-DIO-mCherry (50459-AAV8, Addgene, gift from Bryan Roth), AAV8/rg-hSyn-Cre-dTomato (107738-AAV8/rg, Addgene, gift from Rylan Larsen), AAVDJ-CMV- DIO-eGFP (GVVC-AAV-12, Stanford University Gene Vector and Virus Core), or AAVDJ-CMV-DIO-eGFP-2A-TeNT (Cre-Dependent Tetanus Toxin, GVVC-AAV-71, Stanford University Gene Vector and Virus Core) is delivered bilaterally into DH (1.8 mm posterior, 1.2 mm lateral, 2.25 mm ventral to bregma) and/or RSP (1.8 mm posterior, 1.2 mm lateral, 1.1 mm ventral to bregma) using a Hamilton neurosyringe connected with microinfusing pump (UMP-3, WPI, Sarasota, FL, USA). For each site 0.5 μL AAV solution (2 × 10^12^ GC/mL) was delivered at 0.15 μL/min, and syringes were left in place for 5 min after the injection. Recombinant HAPLN1 (2608-HP-025, R&D Systems, Minneapolis, MN, USA, 100 ng/μL in PBS), TGFB1(763102, BioLegend, San Diego, CA, USA, 10 ng/μL in PBS) or TGFB2 (7346-B2–005, R&D Systems, 10 ng/μL in PBS) at was injected bilaterally into RSP (0.5 μL/site) as described above.

### Immunohistochemistry

Mice were anesthetized with an intraperitoneal injection of 240 mg/kg Avertin and transcardially perfused with ice-cold phosphate buffer followed by 4% paraformaldehyde in phosphate buffer (pH 7.4, 50 mL each per mouse). Brains were removed and post-fixed for 24 hours in the same fixative and then cryoprotected by immersing in 20% and 30% sucrose in phosphate buffer for 24 hours each. After that the brains were frozen in OCT embedding medium and 50 μm freezing microtome sections were made for use in free-floating lectin histochemistry or immunohistochemistry. Biotinylated WFA (1:1000 B-1355–2, Vector Laboratories, Newark, CA, USA) was used to visualize PNN. For immunohistochemistry, primary antibodies against aggrecan (1:1000, ab3778, Abcam, Cambridge, UK), neurocan (1:2000, AF5800 R&D systems), brevican (1:500, 19017–1-AP, Proteintech,Rosemont, IL, USA), phosphacan (1:500, sc-33664, Santa Cruz, Dallas, TX, USA), PVALB (1:5000, pvg-213, Swant, Burgdorf, Switzerland), SST (1:1000, MAB354, Millipore, Burlington, MA, USA), VIP (1:1000, 20077–1513001, Immunostar, Hudson, WI, USA), NPY (1:1000, 22940–1628001, Immunostar), CR (1:4000, CG1, Swant), Iba1 (1:1000, ab5076, Abcam), Olig2(1:2000, ab109186, Abcam), PDGFRa (1:2000, AF1062, R&D Systems), and s100b(1:2000, 287108, Synaptic Systems, Goettingen, Germany) were used followed by corresponding secondary antibodies (1:500, Alexa Fluor conjugated, Jackson IR, West Grove, PA, USA or 1:200, Biotinylated, Vector Laboratories). When using biotinylated systems, the signals were visualized with the ABC-HRP Detection Kit (PK-6100, Vector Laboratories) and TSA Fluorophores (Akoya Biosciences, Marlborough, MA, USA).

### Microscopy, image analysis and quantification

For large scale analyses, sections were scanned using Axioscan Z1 microscope (Zeiss, Oberkochen, Germany) scanner under 10x objective. Images were stitched and converted to TIFF format. RSP findings were additionally verified following confocal microscopy under 60x objective (Fluoview FV10i-LIV, Olympus, Hachioji, Tokyo, Japan). All quantifications were performed with ImageJ using two methods: the multi point plug-in and analyze particles plug-in. Intensity was calculated by measuring the gray value after thresholding to eliminate background interference. Cell counting and gray value analysis were performed in a 0.1 mm^2^ area for each ROIs. The measurements were averaged from 2 sections per animal; each animal was treated as an independent sample for statistical analysis.

### Statistical analysis

Statistical power to detect anticipated effect sizes was determined using power analysis (calculator at http://www.stat.ubc.ca/~rollin/stats/ssize/n2.html) conducted on representative samples of previous work and pilot experiments. For all proposed experiments, minimum power is set at 0.90 to detect an α = 0.05 (two-sided test) for a difference in means from 20% to 40%, with a 15% common standard deviation. Mice are randomly assigned to groups. However, to prevent litter effects, mice from the same litter were assigned to different experimental groups. Viruses and proteins were injected by experimenters aware of the construct, but the mice were then assigned coded numbers by the laboratory technician. The code was available after quantification and before analyses. Statistical analyses were performed using GraphPad Prism. Outlier mice exhibiting abnormal behavior performance determined by Grubbs’ test or with misplaced infusion were excluded. One-way ANOVA or Two-way ANOVA followed by indicated post hoc tests were used for comparisons of three or more experimental groups (only when ANOVA was significant) whereas Student’s t-test was used for comparison of two experimental groups. Homogeneity of variance was confirmed with Levene’s test for equality of variances. Repeated measures (RM) ANOVAs with Geisser Greenhouse correction were performed for within-subject designs. On indicated data, we performed correlation analyses and report Pearson’s r coefficients. All comparisons were conducted using two-tailed tests and the P value for all cases was set to <0.05 for significant differences. Data are expressed as mean ± s.e.m. Statistically significant differences are indicated as *P < 0.05, **P < 0.01, and ***P < 0.001.

## Results

### PNN numbers in the RSP-DH circuit from early to late adolescence

**Figure 1:**
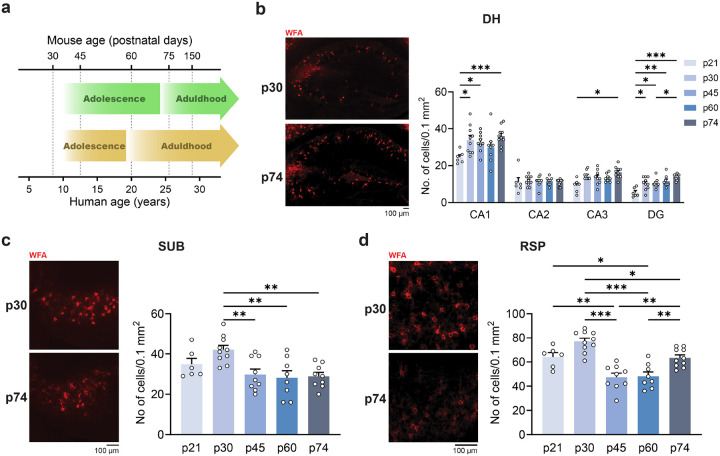


The alignment of mouse and human ages currently (green) and previously (yellow) considered as adolescence is depicted in Fig. 1a. Our findings were interpreted in the framework of the latest considerations.

We quantified the levels of PNN labeled with the *Wisteria floribunda agglutinin* (WFA) in the DH, in the dorsal subiculum (SUB, which provides DH excitatory inputs to RSP^26^), and in RSP from pre-adolescence to late adolescence (p21-p75). The number of PNN in DH (Fig. 1b) remained stable in CA2 and even increased with age in CA1 (p21 vs. p30: p = 0.029, p21 vs. p45: p = 0.017, p21 vs. p75: p < 0.001,), CA3 (p21 vs. p75: p = 0.016), and dentate gyrus(p21 vs. p30: p = 0.032, p21 vs. p45: p = 0.019, p21 vs. p60: p = 0.010, p21 vs. p75: p < 0.001, p45 vs. p75: p = 0.020). On the other hand, in SUB and RSP of both males and females (Fig. 1c, d, Supplementary Fig. 1), PNN numbers were high at p21 and p30, then significantly decreased between p45 and p75 (Fig. 1c: SUB, p30 vs. p45: p = 0.009, p30 vs. p60: p = 0.004, p30 vs. p75: p = 0.005; Fig. 1d: RSP, p21 vs. p45: p = 0.009, p21 vs. p60: p = 0.019, p30 vs. p45: p < 0.001, p30 vs. p60: p < 0.001, p30 vs. p75: p = 0.014, p45 vs. p75: p = 0.004, p60 vs. p75: p = 0.009).

These findings suggested that RSP reached a certain degree of circuit stability before early adolescence (p21), but as the DH progressively matured through adolescence, RSP demonstrated indices of destabilization, such as PNN reduction.

### RSP PNN-associated lectican levels from early to late adolescence

**Figure 2:**
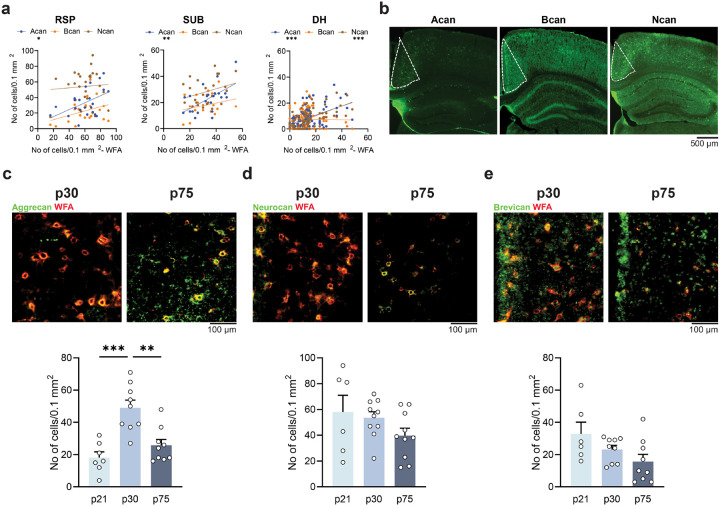


While WFA labels PNN by binding to terminal N-acetylgalactosamine residues of chondroitin sulfate chains of lecticans, it does not inform about the lectican core protein composition, such as aggrecan, neurocan, brevican, and phosphacan, which are important for PNN function^43^. Quantification of the levels of RSP lecticans associated with PNN was first validated showing a significant correlation between WFA and aggrecan in RSP (R^2^ = 0.229, p = 0.002) and SUB (R^2^ = 0.337, p = 0.005) and with aggrecan (R^2^ = 0.251, p < 0.001) and neurocan (R^2^ = 0.349 p < 0.001) in DH (Fig. 2a). Age-dependent analysis revealed a profound and persistent decrease of aggrecan (Fig. 2c: p30 vs. p75: p = 0.001, Supplementary Fig. 2a) and neurocan (Fig. 2d, Supplementary Fig. 2b: male: p30 vs. p75: p = 0.017, female: p21 vs. p75 p = 0.046) in PNN of both p75-old males and females relative to pre-adolescent (p21) or early adolescent (p30) mice. Brevican levels were also reduced in females (Fig. 2e, Supplementary Fig. 2c: p30 vs. p75: p = 0.040). In addition, we observed decreased phosphacan expression in RSP (Supplementary Fig. 3: male: p21 vs. p30: p < 0.001, p21 vs. p75: p < 0.001, p30 vs. p75: p < 0.001; female: p21 vs. p30: p < 0.001, p21 vs. p75: p < 0.001). Although lecticans in the DH also showed some age-related changes, they mainly involved increased incorporation of aggrecan and neurocan in PNN (Supplementary Fig. 4a, age effect: Acan male: p = 0.011, Acan female: p = 0.039, Ncan male: p < 0.001), whereas SUB showed similar decreases as RSP (Supplementary Fig. 4b, age effect: Acan male: p =0.022, Acan Female: p < 0.001, Ncan male: p = 0.036). Phosphacan (age effect: DH: male: p < 0.001, female p = 0.001, SUB: male: p = 0.003, female p < 0.001) and brevican (in females only, DH: p < 0.001, SUB: p = 0.006) showed age-related decrease in all extracellular matrix compartments.

The late adolescent decrease of lectican levels was consistent with the decrease of PNN numbers during adolescence. However, although PNN levels similarly decreased in males and females, the levels and developmental changes of individual lecticans (neurocan in males and brevican in females) suggested a sex-specific regulation of ECM functions.

### RSP cellular composition from early adolescence to middle adulthood

**Figure 3:**
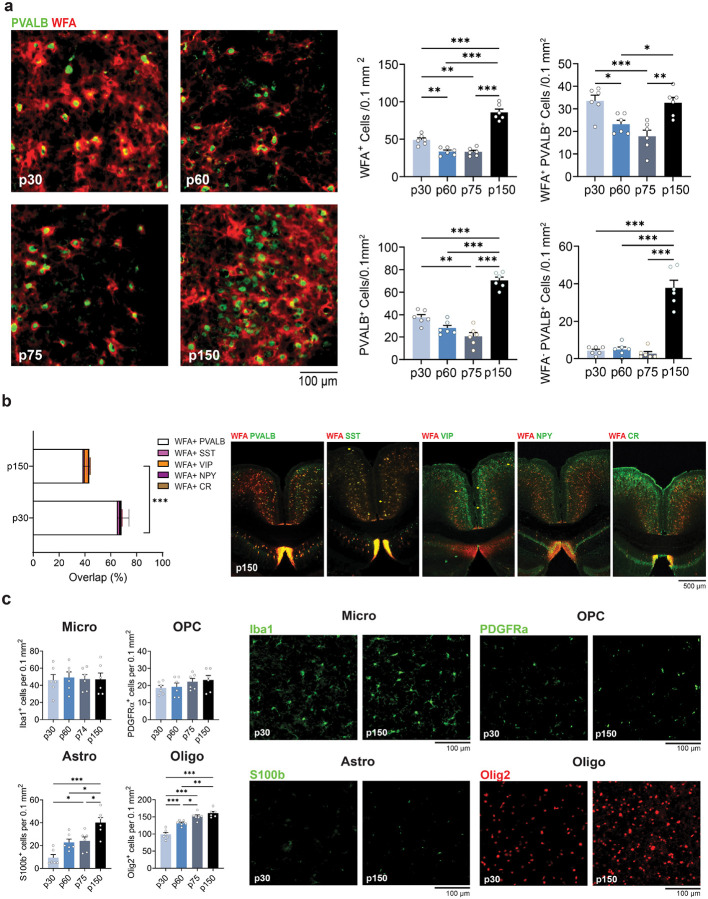


Given the predominant RSP PNN localization around PVALB-positive interneurons (Fig. 3), we next sought to determine whether the decrease of PNN might be related to changes of the numbers of interneurons or other cell types that control lectican secretion and PNN degradation. For this study, we extended the period of analysis to middle adulthood to also determine whether the changes occurring in late adolescence are transient or persistent. Quantification revealed that the number of PVALB interneurons closely paralleled the changes of PNN, showing a significant decrease at p75 (WFA: p = 0.006, PVALB: p = 0.002 vs. p30)., followed by a large increase by p150 (WFA and PVALB: p < 0.001 vs. all other groups, Fig. 3a). Interestingly, the proportion of PVALB-positive and WFA-negative interneurons as well as PVALB-negative and WFA- positive interneurons at p150 was substantial (~29%). This was not due to the formation of PNN around other main interneuron classes including interneurons showing somatostatin, neuropeptide Y, vasoactive intestinal peptide, and calretinin labeling (Fig. 3b, p30 vs. p150: PVALB: p = <0.001 SST: p= 0.999, VIP: p = 0.690, NPY: p > 0.999, CR: p > 0.999). The reduction of the number of PVALB-positive interneurons was specific for this population and did not involve similar changes of other cell types, such as microglial fluctuations found in the prefrontal cortex^44^, although in middle adulthood, the number of S100b-positive astrocytes (which are predominant in RSP) and oligodendrocytes also increased (Fig. 3c, age effect on microgila: p = 0.988, oligodendrocytes precursor cells: p = 0.366, astrocytes and oligodendrocytes: p < 0.001).

### Expression of early adolescent context memories through middle adulthood

**Figure 4:**
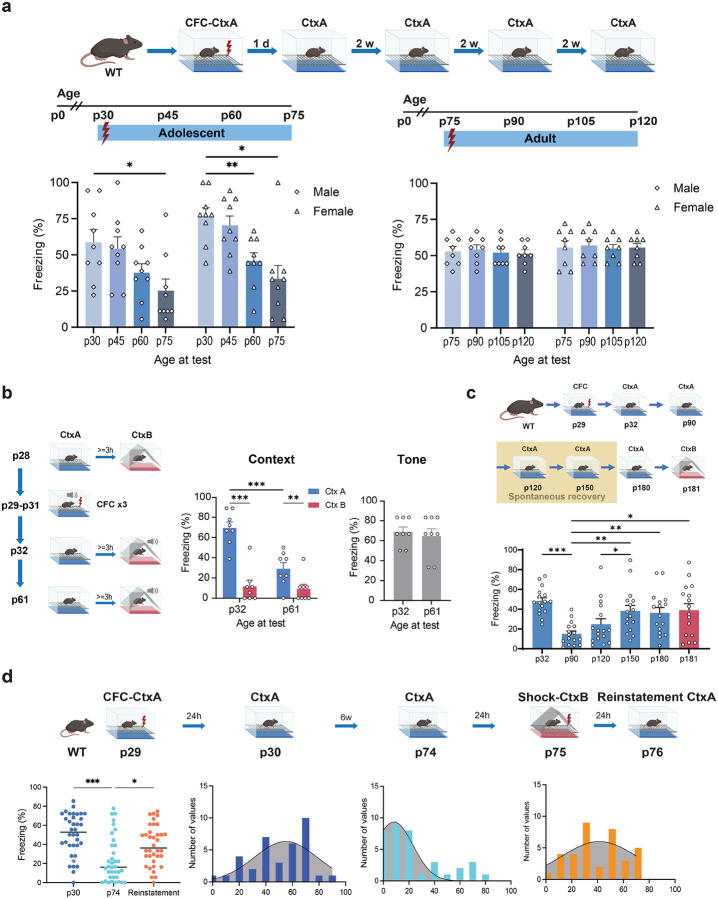


We next examined whether the observed RSP changes affect the developmental features of CFC memory formation, expression, generalization, spontaneous and shock-induced recovery, throughout early adolescence and middle adulthood. We first trained male and female mice in a one-trial contextual fear conditioning paradigm (CFC, 3 min CtxA exposure, 2 sec footshock, 0.7mA, constant current) on p29 or p75 and tested every two weeks thereafter for memory expression by determining freezing behavior in response to context re-exposure. Mice of both sexes trained on p29 showed impaired CFC memory by p75, as revealed by a significant decrease in their freezing levels (age at test effect: p < 0.001), whereas mice trained at p75 retained their memories within a similar 6-week time frame by p120 (age at test effect: p = 0.744, Fig. 4a). This effect was replicated in a follow-up experiment whose objectives were threefold: (i) determine whether the impairment might have been due to enhanced extinction induced by repeated testing; (ii) whether the adolescent memory lacked specificity that might have affected its persistence; and (iii) whether hippocampus-independent associative memories undergo similar delayed impairments. To address these questions, we exposed male and female mice on p28 to modified fear conditioning paradigm allowing for determination of context specificity/discrimination^45, 46^ and for assessment of both context (CFC) and delay tone-dependent fear conditioning (dTFC, Fig. 4b, left). The paradigm consisted of a 3-min pre-exposure to CtxA and CtxB. Mice were then fear conditioned to context and tone by exposing them to CtxA (3 min), tone (30 sec), and footshock (2 sec) with the same parameters described above, once a day over 3 days. Memory tests performed one day (p32) or one month later (p61) also demonstrated a significant impairment of adolescent context-shock memory (Fig. 4b, middle, p32 vs. p61 in CtxA: p < 0.001) without affecting the tone-shock memory (Fig. 4b, right, p = 0.516). At all ages between p25 and p65, mice showed an intact ability to differentiate between the conditioning context (CtxA) and context where shock was never delivered (CtxB) (p32: p < 0.001 p61: p = 0.036, also see Supplementary Fig. 5). Interestingly, although during late adolescence mice were unable to recall previously acquired context memories, mice trained during late adolescence were able to form long-lasting new context memories, ruling out that the observed impairments could have been related to impaired fear or anxiety expression.

Subsequent experiments were devised to determine whether the observed memory deficits reflected a problem of memory degradation, retention, or access. We first extended the period for memory determination through adulthood seeking to determine whether the context memory might recover with the increase of PNN numbers around middle adulthood. While this was indeed the case with respect to the re-emergence of freezing behavior, the recovered memory was no longer context specific, as revealed by similar freezing in previously shock-associated CtxA and novel context CtxB (p30 vs. p90 p < 0.001, p90 vs. p150: p = 0.002, p90 vs. p180: p = 0.012, p180/CtxA vs. p181/CtxB: p = 0.997, Fig. 4c). To alleviate the confounding effects of generalization found with the passage of time among similar contexts^47^, we used dissimilar contexts that did not show time-dependent generalization (Supplementary Fig. 6).

We next examined the retention of adolescent memories by determining their proactive interference with the formation of CtxB memories where mice re-experienced the same footshock. The goal was to determine whether memories can be reinstated before middle adulthood and PNN build-up. To this end, we performed two studies. In the first study, we investigated memory reinstatement as a function of the aversive experience by using a no-shock control. A second study was devised to provide a replicate of the reinstatement effect, but after retroactive interference of enriched environment (EE)^48^, which has established anxiolytic effects^49^. Data from both studies were presented together in Fig. 4d, whereas findings for each experiment separately are shown in Supplementary Fig. 7. Overall, the findings replicated the adolescent memory expression deficit (p30 vs. p74: p < 0.001). but showed a significant generalization of freezing behavior between CtxA and CtxB after CtxB CFC (p74 vs. p76 reinstatement test: p = 0.014 Fig. 4d, bottom left), consistent with the spontaneous recovery finding (Fig. 4c), further ruling out memory loss. Moreover, even after exposing the mice to EE which additionally reduced CtxA fear expression (p30 vs. p74: HC and EE: p < 0.001, EE vs. HC: p44: p = 0.004, p60: p = 0.037), the CtxA memories were retained, as revealed by their robust reinstatement (EE: p74 vs p76 CtxA: p < 0.001, Supplementary Fig. 7b).

Together, these findings demonstrated that although stress-induced context memories acquired during early adolescence are initially robust and specific, they showed delayed expression deficits (relative to memories acquired during adulthood), coinciding with the decrease of RSP PNN and PVALB interneuron numbers. The impairment was sex-independent and not due to memory loss, inability to express fear, or general impairments in retrieving negative associative memories.

### Effect of RSP PNN stabilization on cellular and memory impairments during adolescence

To determine whether the late adolescent reductions of context memory expression and PVALB interneuron numbers were directly linked to PNN degradation, we next investigated whether these changes can be prevented by stabilizing PNN using RSP-targeted injections of hyaluronan and proteoglycan link protein 1 (HAPLN1, Fig. 5a). This approach was selected based on its documented efficacy of HAPLN1 to stabilize the ECM in various *in vivo* models^50, 51^. Injections of HAPLN1 alleviated the impairments of adolescent memory expression (Age of test × HAPLN1 interaction: p = 0.032, p32 vs. p60: Veh: p = 0.002, Hapln1: p = 0.060; p60: Veh vs Hapln1: p=0.038), the degradation of PNN (p < 0.001), and the reduction of PVALB interneuron numbers (p = 0.021). relative to control, vehicle (Veh)-injected mice (Fig. 5b, c). Given that HAPLN1 acts through TGFB signaling^52–54^, which enhances lectican gene expression^53, 55^, we also examined whether RSP injection of TGFB1 or TGFB2 can also attenuate PNN degradation and rescue the memory impairments. Unlike HAPLN1, a single infusion of TGFB1 and TGFB2 on p45 failed to produce a long-lasting behavior effect (Infusion × Age of test interaction: p = 0.205, Fig 5d) despite transiently attenuating the PNN reduction (7d after infusion, p = 0.039, Fig 5e). This could be contributed by a shorter half-life or action duration of TGFB compared to HAPLN1. Therefore, we performed a more robust and sustained manipulation of TGF-beta signaling by injecting an AAV vector carrying Cre recombinase (AAV-Syn-Cre) into the RSP of TGFBR2-floxed mice on p31, introducing a RSP-targeted neuron-specific knockout of TGFBR2. Knockout mice demonstrated accelerated memory decline (AAV × Age of test interaction: p = 0.045, p45 mCherry vs. p45 Cre: p = 0.010), PNN degradation (p = 0.008), and reduced PVALB neuron numbers (p = 0.002). by p45 compared to control (AAV-Syn-mCherry) mice (Fig. 5f). These results revealed that disruption of early-adolescent memory was closely associated with PNN decrease levels during adolescent development. Together, our findings indicated that PNN degradation directly contributed to the loss of PVALB interneurons, identifying them as the most vulnerable cell population with respect to cortical ECM dynamics.

## Discussion

We demonstrated that the RSP circuits undergo extensive molecular, cellular, and structural modifications during late adolescence that support the formation of “adult type” (long-lasting, context-specific) memories while interfering with the expression and fidelity of memories acquired during early adolescence. These findings identify a hitherto unrecognized, late maturation period of the RSP memory circuit with important consequences on memory dynamics.

Similar to our RSP findings, a decrease of PVALB and PNN between p28-p56 was previously reported for the dorsal intermediate entorhinal cortex, but not temporal cortical areas^56^ or the medial prefrontal cortex^57^, indicating that region-specific differences within the cortex itself are to be expected during adolescent cortical reorganization. Indeed, a recently reported late adolescent reorganization (p60) of the mouse prefrontal cortex was predominantly microglia-driven^44^, whereas the RSP reorganization shown here was primarily PNN-driven. This was indicated by the observation that PNN stabilization preserved the number of PVALB interneurons, and previous evidence of compromised PVALB interneuron viability after PNN degradation^58, 59^. Despite these striking mechanistic differences, however, both studies provided evidence for nonlinear cortical reorganization (increased initial maturation/activity during early adolescence followed by destabilization during late adolescence), which underlined the establishment of adult memory phenotypes.

The memory benefits acquired through late adolescent cortical changes appeared to be at the cost of early adolescent memories, which showed significant instability first with respect to expression, and then with respect to generalization. Such findings strongly support the model of generalization rooted in memory instability^60^. However, rather than a cost, impairments of memory expression and emergence of generalization can alternatively be viewed as mechanism with unique adaptive advantages. For example, impaired expression of previously formed memories could facilitate new knowledge acquisition, and context attunement, as it has been proposed for forgetting more generally^61, 62^, whereas generalization enables the application of previous knowledge to new situations^45, 46^. In this way, memories acquired before late adolescence can still shape behavior in later life.

As most previous rodent studies^15, 16, 24, 63^, we saw no evidence of memory suppression^14^. However, the unreliable retrieval of early relative to late adolescent memories, is at odds with the widely held view that memories formed during early adolescence are stable^24, 25^. An important reason for this discrepancy could be that most existing evidence stems from hippocampal research, especially DH, which does not take into consideration cortical control over memory expression as early adolescent memories become less hippocampally- and more cortically-dependent with the passage of time^28, 64–66^. Given that the decrease of RSP PNN numbers parallelled the increase of DH PNN numbers, we suspected that impairments of early adolescent memories might be triggered by the maturation of excitatory DH-RSP connections, establishing dominance of DH control over memory formation, retrieval, and specificity^26, 67, 68^. However, permanent inactivation of synaptic transmission in DH-RSP projections did not prevent the adolescent memory impairments, (Supplementary Fig. 8), suggesting that the observed changes were more likely intrinsic to RSP rather circuit driven, as previously found with PNN dynamics in the visual cortex^69, 70^.

The memory expression deficits were not sensitive to the absolute numbers of PNN and PVALB, but to their decline between the time they were formed (early adolescence) and the time of retrieval tests (late adolescence). Accordingly, memories formed during late adolescence (p60–75) were fully preserved later on although PNN and PVALB levels were lower relative to early adolescence. These findings can be explained by the documented contributions of PNN stabilization to the persistence of existing memories^22, 23, 71^, and PNN destabilization to the formation of new memories^43, 72^. An interesting possibility is that PNN might also modulate memory specificity through the degree of overlap PVALB interneurons, as suggested by the parallel decrease of PNN/PVALB interneuron overlap with the increase of memory generalization after spontaneous recovery. Whereas the restoration of PVALB interneuron numbers is likely due to interneuron generation in the adult mouse cortex^73^, it remains to be established whether the causes of their reduced overlap with PNN lie in the greater fluctuations of PVALB levels between adulthood and middle adulthood^74^, in the progressive build-up around excitatory cortical neurons^75, 76^, or other mechanisms.

The late adolescent changes of the extracellular matrix (PNN numbers, lectican levels) are also noteworthy because genetic variants contributing to the greatest phenotypic variability across mouse strains are most significantly enriched in extracellular matrix pathways^77^. This suggests that the expression of adolescent memory may vary with individual genotypes. Indeed, even within a genotype we observed individual variability, nevertheless, the memory instability phenotype was replicated in most mice (~70%) and in three genotypes [C57BL6N, floxed TGFBR2, and NT-GFP (mixed FVB/N × C57BL/6J)], suggesting a robust phenotype.

Overall, our findings suggest a more subtle contribution of endogenous PNN fluctuations to memory functions relative to exogenously applied enzymatic PNN and ECM degradation^22^. We propose two potential mechanisms by which such fluctuations could impair memory expression. One is a transient failure to reconstruct the memory-related neuronal activity patterns due to RSP circuit instability, a problem that could be partly overcome by re-experiencing the stressful event in a different context, but at the expense of context specificity. Additionally (or alternatively), RSP PNN fluctuations could reorganize memory access, dynamically prioritizing or restricting access to memories based on their valence or recency. Either scenario would support a view of memory as a reconstructive process, which involves memory modification and loss of detail rather than re-excitation of fixed and fragmentary traces^78, 79^, a view that is gaining increasing support from electrophysiological^80–82^ and cellular approaches^83^.

In addition to aligning with human research on adolescent cortical reorganization, several memory features observed in our study support their translational relevance. The memory loss found in late adolescence is in line with the decrease of the associative structure of episodic memories between early and late adolescence^84^, whereas the memory recovery shows striking similarities with the “reminiscence bump” phenomenon in human episodic memory research, which refers to the tendency of adults to recall a disproportionately large number of memories from their adolescence and early adulthood^85, 86^. One similarity is the timing of adolescent memory formation (early adolescence) and memory expression (middle age). Others include the retention of the stress-related memory component (revealed by freezing behavior) but loss of context specificity, which resembles the ease of remembering emotional memory content but impaired memory for contextual detail in human reminiscence bump^87^. All these changes of memory expression and specificity coincided with the endogenous or pharmacologically and genetically induced fluctuations of PNN/PVALB numbers, suggesting that ongoing ECM reorganization in posterior cortices might also contribute to the human memory dynamics. Abnormalities of ECM dynamics during this late RSP maturation period, on the other hand, could increase disease risk of adult onset mental illnesses, such as schizophrenia and major depression, especially in carriers of high risk ECM gene variants^88^. Such abnormalities could contribute to the episodic^89, 90^ (especially contextual^91^) memory deficits, default mode network dysfunctions^39^, and extracellular matrix abnormalities^92–94^ reported for these illnesses.

## Supplementary Material

Supplementary Files

This is a list of supplementary files associated with this preprint. Click to download.

- Supplementaryinformation.docx

## Figures and Tables

**Figure 5:**